# The Treasury of Wharton's Jelly

**DOI:** 10.1007/s12015-021-10217-8

**Published:** 2021-10-13

**Authors:** Rebecca Guenther, Stephan Dreschers, Jessika Maassen, Daniel Reibert, Claudia Skazik-Voogt, Angela Gutermuth

**Affiliations:** 1 Department for Applied Cell Biology, Fraunhofer Institute for Production Technology, Steinbachstr. 17, 52074 Aachen, Germany; 2 Clinic for Gynaecology, University Hospital Aachen, Pauwelsstr 30, 52074 Aachen, Germany

**Keywords:** Umbilical cord tissue, Multipotency, Pluripotency, Very small embryonic like stem cells, Regenerative medicine, Wharton's jelly, Trilineage differentiation.

## Abstract

**Background:**

Postnatal umbilical cord tissue contains valuable mesenchymal progenitor cells of various differentiation stages. While mesenchymal stem cells are plastic-adherent and tend to differentiate into myofibroblastic phenotypes, some round cells detach, float above the adherent cells, and build up cell aggregates, or form spheroids spontaneously. Very small luminescent cells are always involved as single cells or within collective forms and resemble the common well-known very small embryonic-like cells (VSELs). In this study, we investigated these VSELs-like cells in terms of their pluripotency phenotype and tri-lineage differentiation potential.

**Methods:**

VSELs-like cells were isolated from cell-culture supernatants by a process that combines filtering, up concentration, and centrifugation. To determine their pluripotency character, we measured the expression of Nanog, Sox-2, Oct-4, SSEA-1, CXCR4, SSEA-4 on gene and protein level. In addition, the cultured cells derived from UC tissue were examined regarding their potential to differentiate into three germ layers.

**Result:**

The VSELs-like cells express all of the pluripotency-associated markers we investigated and are able to differentiate into meso- endo- and ectodermal precursor cells.

**Conclusions:**

Umbilical cord tissue hosts highly potent VSELs-like stem cells.

**Graphical Abstract:**

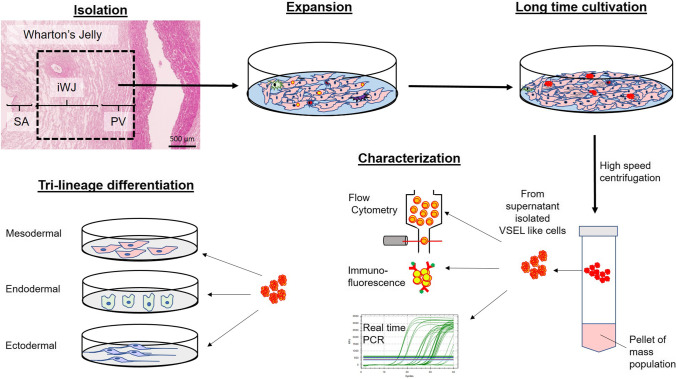

**Supplementary Information:**

The online version contains supplementary material available at 10.1007/s12015-021-10217-8.

## Introduction

Neonatal mesenchymal stem cells from umbilical cord tissue (UC-MSCs) are an attractive stem-cell source for regenerative medicine [1]. In contrast to the most commonly utilized mesenchymal stem cells (MSCs) from bone marrow or adipose tissue, these cells are not influenced or diminished in their function through stress that cells experience in the ageing body [2–4]. When cells age, the activities and functions of tissue-derived MSCs decline, which is regarded as a major challenge for MSC therapies and tissue engineering [5, 6]. Difficulties like these can be excluded for UC-MSCs, and *in vitro* it could be shown that this neonatal multipotent stem-cell source has a stronger proliferation capacity [7] than that of MSCs from aged-tissue sources; furthermore, their morphology is stable for a longer period of time during passaging [6, 8]. What is also remarkable is that UC-MSCs continue to be immune-privileged following their differentiation into multiple lineages [9]. Up to now, UC-MSCs had been investigated in depth in several clinical trials and research projects that addressed various clinical pictures [10, 11]. In summary, in all studies the application of UC-MSCs resulted in an improvement of the respective health situation, while the UC-MSCs do not form teratoma [1], initiate immune reactions or show unexpected side effects. In terms of the mode of action, the common view of the most researchers is that UC-MSCs positively influence healing processes through their immunoregulatory and immunosuppressive functions based on the secretion of soluble messenger compounds or on direct cell-to-cell contact [12–14]. Due to insufficient traceability, the cautious estimation for *in vivo* applications is that UC-MSCs indirectly contribute to tissue build-up [15]. In contrast, many have documented, *in vitro* for UC-MSCs as well as for MSCs, their ability to differentiate into different tissues of mesenchymal lineages such as adipogenic, chondrogenic and osteogenic lineage cells [16]. Clonal analysis has recently revealed that MSCs are a heterogeneous mixture of cells that differ in their stage of lineage commitment and extent of differentiation, and that only a fraction of plastic-adherent cells dispose of colony-forming-unit (CFU) potential and exhibit multipotency [17]. With regard to UC-MSCs, lineage commitment means a development towards Wharton’s Jelly (WJ) or perivascular residing cells that partially resemble mesenchymal fibroblasts, but similar to smooth muscle cells, express fibers and have contractile properties [18]. It is well known that MSCs directly experience lineage commitment by cultivation on plastic surfaces and FBS-containing media and that UC-MSCs differentiate thereby into a myo-fibroblastic phenotype. This phenotype is alpha smooth-muscle actin (α-SMA) positive and has large flattened cell shape and visible stress fibers [19]. However, a small degree of UC-MSCs seem to be uninfluenced by external cell-culture conditions and, instead of differentiating into myo-fibroblastic like cells, they are able to form spheres spontaneously from the adherent cell layer. [20, 21]. In addition, a small proportion of UC-MSCs have been shown to express pluripotency-associated markers and to have the intrinsic capacity to trans-differentiate into cells of foreign germ-layer origin [17, 20, 22], for example into cardiomyocytes [23], skeletal muscle cells [24], cholinergic neurons [24], hepatocytes [25, 26], or pancreatic islet-like cell clusters [12]. Some research has already attempted to characterize a kind of more naïve cell with higher stem cell potential within the MSC mass population (MP). In this context Kuroda et [27] could detect MSC populations of CD105/SSEA-3 positive adult human stem cells in adult bone marrow or skin, which can generate cells with the characteristics of the three germ layers from a single cell. Recently, Leng Z also discovered this CD105/SSEA-3 positive stem-cell population in UC-MSCs [28], but although these cells showed a strong ectodermal differentiation potential, they came, in contrast to their counterparts, from bone marrow or skin and were not differentiable into endo- or mesodermal lineages.

In this study, we looked further for cells with extended stem-cell properties and discovered a small population of small round luminous cells with pluripotent properties which were always visible in companionship with the MSC-MP in Passage 0 directly after tissue outgrowth. Since these cells diminish with each medium exchange, we collected these small round shaped cells and re-injected them into the culture plates. We could observe that the small cells multiplied through the co-culture with the aging UC-MSC MP, so that in the end we could enrich enough cells to measure expression of pluripotency markers and their ability to differentiate into three germ layers.

## Results

### Cell Morphology

**Fig. 1:**
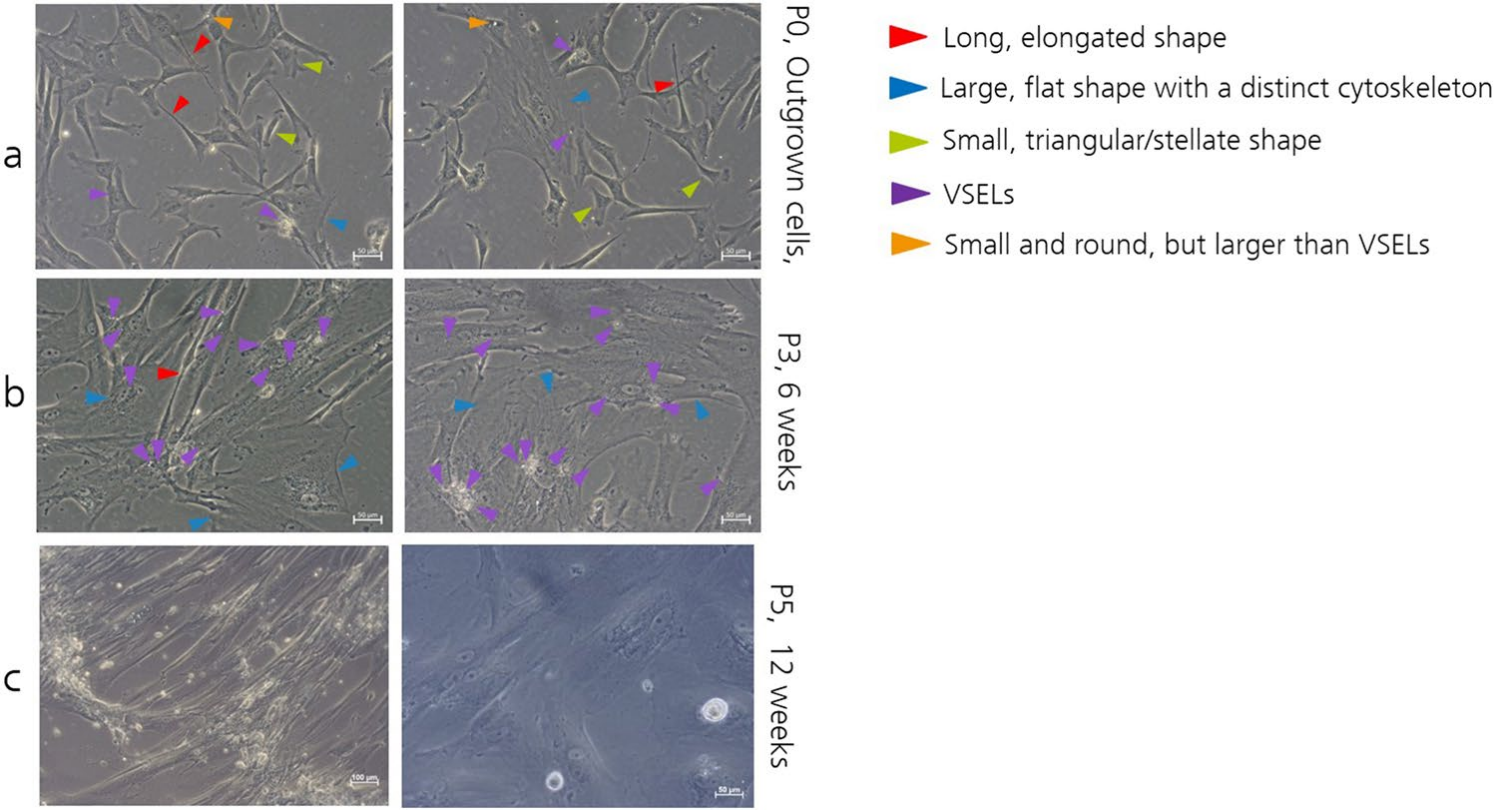
Morphology of UC tissue cells in relation to cultivation time. Cells were obtained by cell outgrowth from WJ tissue pieces and displayed of different morphologies as long, elongated shape, large flat, small triangular/stellate, small round or tiny round (**a**). Scale In passage three (round about 6 weeks after outgrown) the cells were less heterogeneous and mostly the flat or elongated and tiny round cells were represented (**b**). After long-term cultivation of more than 12 weeks, only large elongated cells remain, with many single, dubletts or aggregates of tiny round cells lining the surface (**c**, left) or tiny cell developed spheroids, swimming in the supernatatns of large cells. (**c**, right). Scale bar for all represents 100 μm with the exception of (**c**, left) which is 50 μm

UC-MSCs were derived from the WJ by outgrowth from tissue pieces on uncoated plastic surface. As demonstrated in Fig. 1, the type of cell morphology of WJ-derived cells varied with each passage and cell density. Directly after tissue outgrown, cells of Passage 0 cells are of various morphologies. The arrows show exemplary cells with different shapes, as to be named small triangular/stellate, large flat cytoskeleton, long and elongated, small round cells and also to a little portion of tiny round luminous cells. Figure 1b demonstrates that the composition of the cell types is different in Passage 3, and that mainly large flat or elongated cells can be detected, while hardly any of the small round or triangular ones are left, and the tiny luminous ones have become more numerous. It looks like the tiny cells stick to the large cells. Some of the large one display stress fibers and look like myo-fibroblasts. In comparison to the data from the Kucia et al. [29], these tiny cells with a diameter between 5 and 7 μm looked like very small embryonic like stem cells (VSELs). After long term cultivation of 12 weeks we could observe that these very small cells began to multiply and either collected into spheroids with a diameter of 20 to 80 (Fig. 1c, right) µm or remain as single, dubletts or cell aggregates in the cell culture supernatants (Fig. 1c, left).

### FACS

**Fig. 2:**
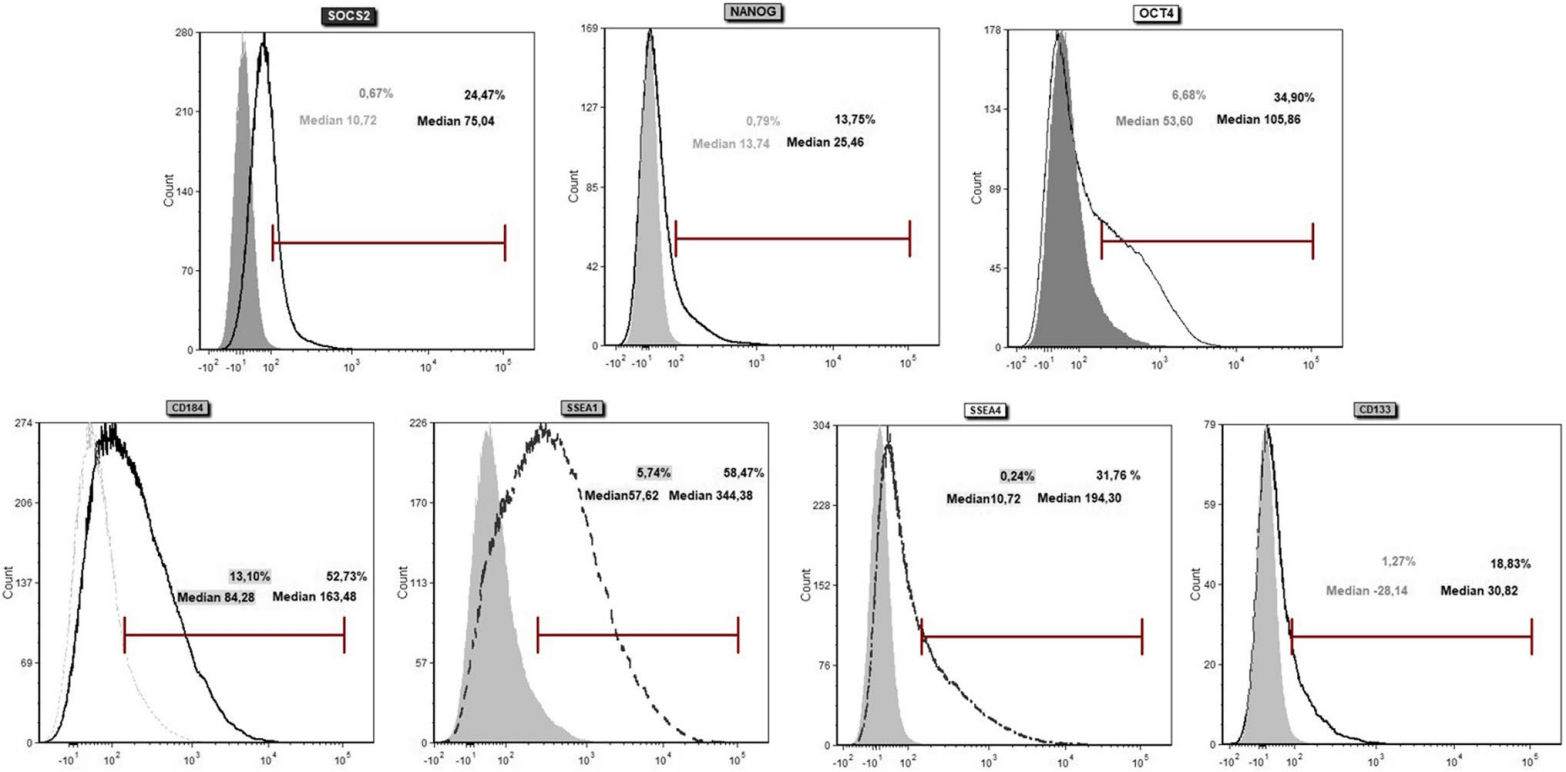
Flow cytometry. A typical example of FACS based expression analysis for transcription factors (upper histograms) and surface receptors (lower histograms) of VSELs. Each histogram compares given antibodies (given in black numbers) and specifc isotype controls (given in red numbers). Percentage of positive VSELs and the median fluorescence were assessed

As demonstrated on Fig. 2, the tiny luminous cells (VSELs) were measured for pluripotent surface marker expression CD184, SSEA-1 and CD133 as well as for the transcription factors Sox-2, Nanog and Oct-4. The samples were strongly contaminated with cell fragments that could not be removed by centrifugation, causing a high background signal. Nevertheless, we measured a population with the adequate size of VSELs that express all pluripotency marker. After subtraction of the isotype controls, we measured 24.47 % Sox*-*2, 13.75 % Nanog, 34.30 % Oct-4 positive cells with a corresponding mean fluorescence intensity of 75.04, 25.46, 105.86, respectively. The proportion of CD184, SSEA-1, SSEA-4 and CD133 positive cells were 163.48 %, 58.47 %, 31.74 and 18.83 % with a respective MFI of 163.48, 344.38, 194.30 and 30.82, respectively.

### Gene Expression

**Fig. 3:**
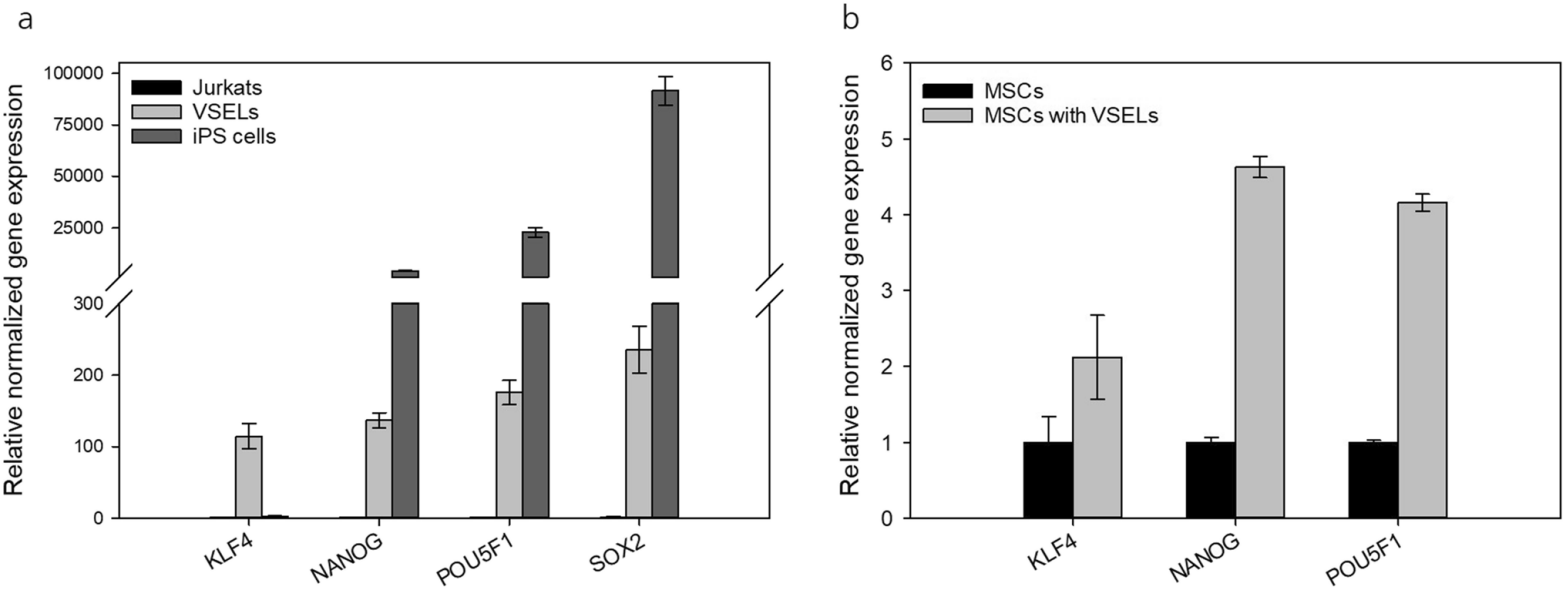
Pluripotency associated marker gene expression of enriched VSELs. Relatively normalized gene expression of *KLF4, NANOF, POU5F1* and *SOX2* in VSELs seperated from UC-MSCs supernatants (dark grey bars) were compared to according gene expression of Jurkat cell line cells (light grey bars) (**a**). Relative normalized gene expression of *KLF4*, *NANOG* and *POU5F1* of UC-MSCs with (dark, grey bars) or without (light grey bars) in supernatant existing VSELs (**b**). Data were normalized by means of the housekeeping genes *GAPDH* and *B2M*

For qPCR analysis, processed VSELs from MSC cell culture supernatants were analyzed regarding their relative normalized gene expression of the pluripotency-associated marker genes *NANOG*, *POU5F1*, *SOX2* and *KLF4*. Jurkat cells were used as negative control as these cells are known to stably express all of these genes and induced pluripotency (iPS) cells were used as positive control. Figure 3a demonstrates that VSESLs express 38.3 times higher *KLF4* than iPS cells. The expression of *NANOG, POU5F1* and *SOX2* were, however, 30-, 129,3- and 387.7-fold higher in iPS as VSELs. In comparison to the negative control, the expression rate for *KLF4, NANOG, POU5F1* and *SOX2* were 114.4-, 13.9-, 176.0-, and 235.0-fold, respectively. Figure 3b demonstrates that addition of VSELs from cell culture supernatants to UC-MSCs enhances the gene expression rate of pluripotency associated markers *KLF4*, *NANOG* and *POU5F1* by 2.1, 4.6 and 4.1-fold, respectively.

### Immunofluorescence

**Fig. 4:**
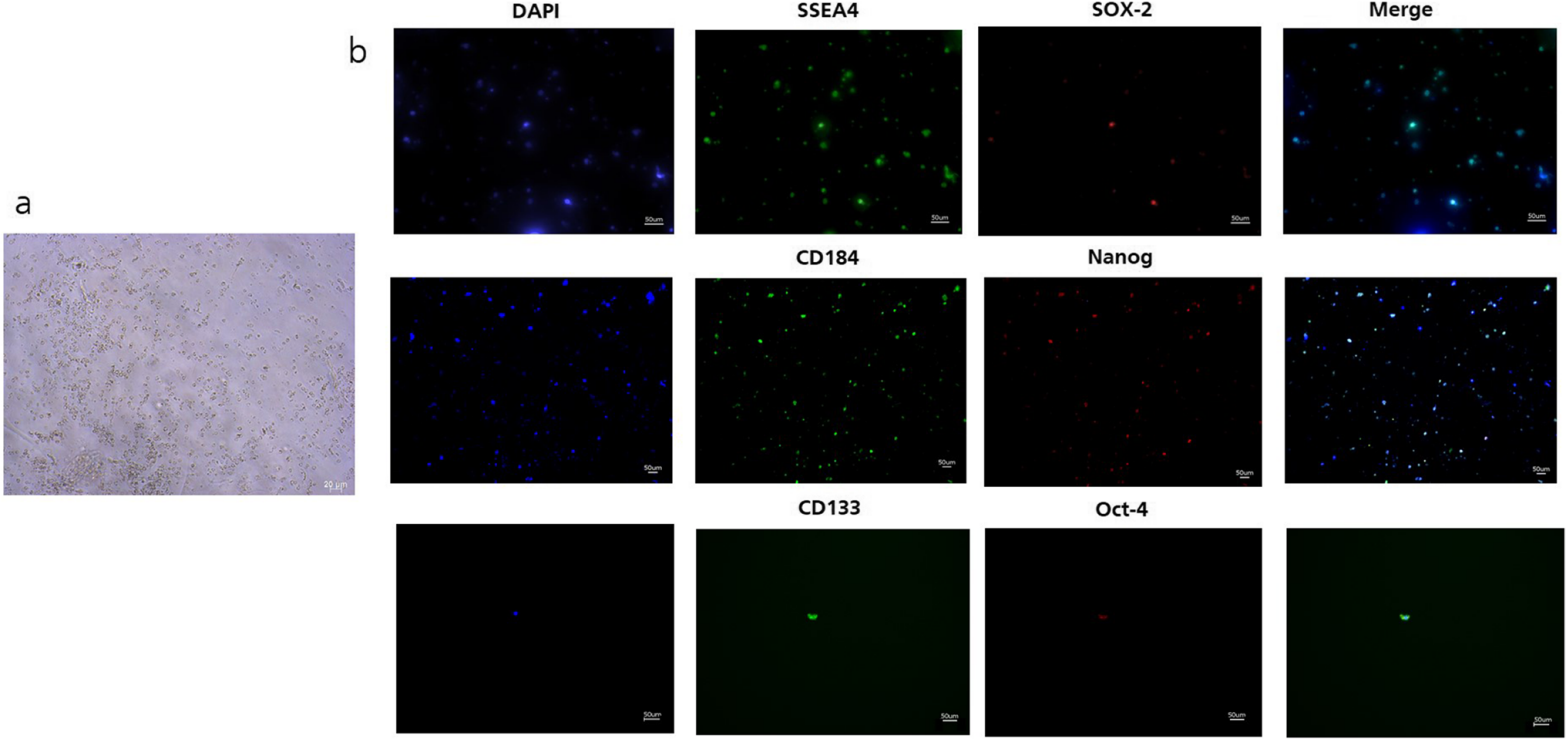
Expression of pluripotency associated marker proteins examined by Immunofluorescence staining. VSELs were separated from UC-MSCs mass population, the tiny cells are between 5 and 7 μm and surrounded with dark spots (**a**). VSEL suspension cells were stained extracellular with anti-bodies against SSEA-4, CD184, and CD133 followed by nuclear staining of transcription factors Nanog, Oct-4, and Sox-2. Expression of each marker was detected. The nuclei were stained with HOECHST. Weak background Fluorescence signals of isotype controls were subtracted. The scale bars represented 50 μm (**b**). The magnification for SSEA-4/Sox-2 and CD133/Oct-4 was 20-fold and for CD184/Nanog 10-fold

For immunofluorescence analysis the small cells were taken from the cell culture supernatants. Therefore, the very small round cells were pooled and purified after sphere dissociation or after the separation procedure. On closer inspection, each cell is surrounded by dark spots on the outer edge of the cytoplasm (Fig. 4a), which can also be seen at isolated VSELs derived from other tissues such as peripheral blood or oocytes [30, 31]. Thanks to immunofluorescence staining, we can provide evidence that isolated very small round cells express several embryonic stem cell markers as Nanog, Oct-4, SOX-2 and SSEA-4, as well as a VSEL-typical marker CXCR-4 (CD184) (Fig. 4b). Double staining of SSEA-4 /SOX-2 revealed that approximately one third of SSEA-4 positive cells were SOX-2 positive. The ratio for CD184/Nanog double stained cells was different as about 90 % of CD184 positive cells were also positive for Nanog. Only a few cells were positive for CD133 in combination with Oct-4 and exemplary we demonstrate one cell of them. The isotype controls for surface and nuclear markers were negative, (supplemental data).

### Tri-lineage

**Fig. 5:**
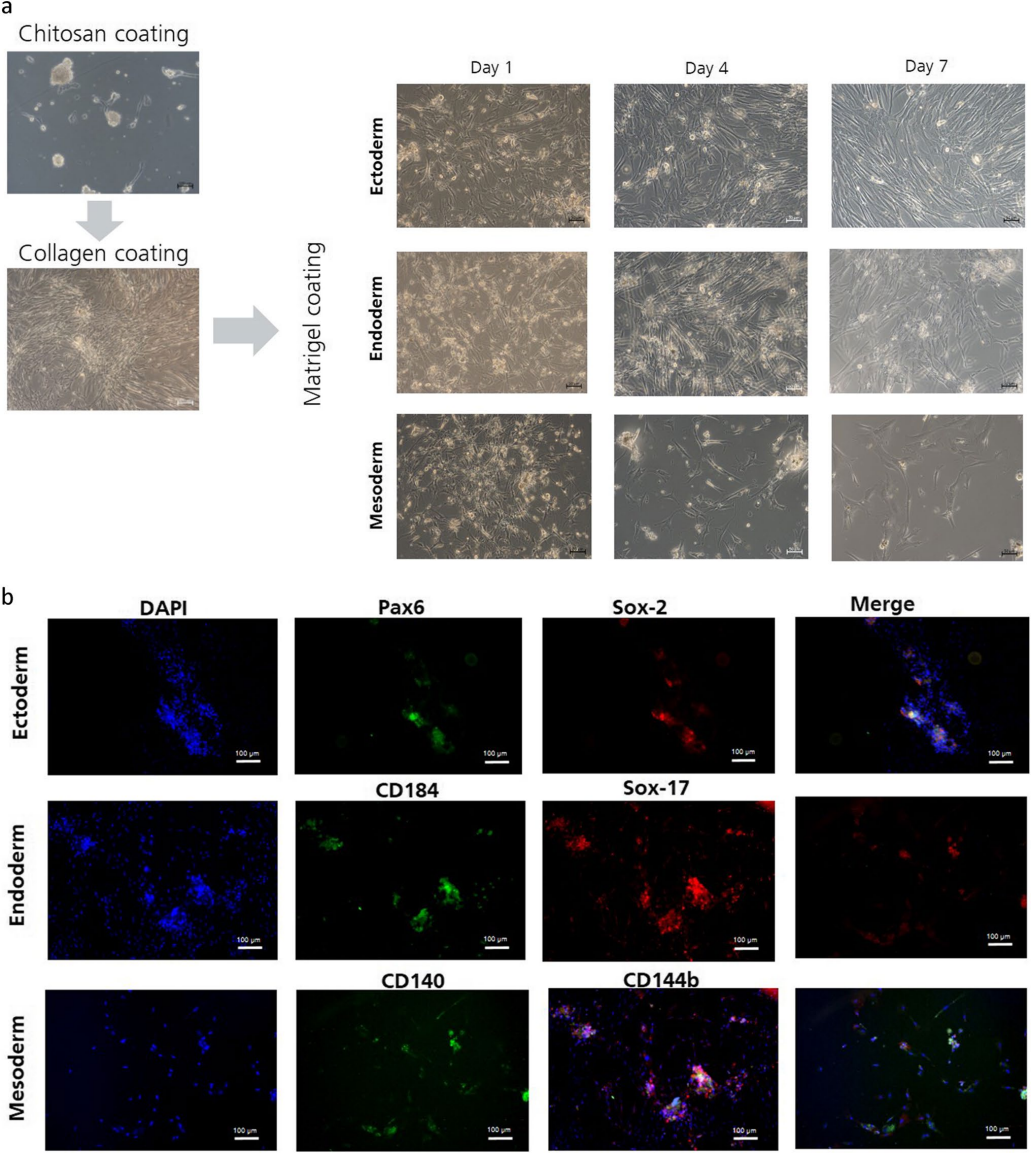
Evaluation of ecto-, endo-and mesodermal differentiation of UC-MSCs spheroids. To evaluate the differentiability into three different germ UC tissue was let grown out on chitosan coated surfaces until UC-MP cells performed spheroids from adhering cells. For multiplication spheroids with a diameter between 80–100 μm or 100–200 μm were seeded on collagen I coated surface until confluence was reached. Adherent cells were trypsinized and seeded onto matrigel coated surface for tri-lineage-differentiation. Phase contrast microscopy images demonstrated the morphology of cells after 1, 4 and 7 days. (**a**) The scale bars represents 50 μm. For confirmation of early differentiation into three germ layers, cells were with stained with appropriate ecto- endo- and mesodermal specific marker Pax6, Sox-2, and Sox-17, CD184 and CD140, CD144b, (**b**). The scale bars represent 100 μm

To determine the potential of UC-MSCs to differentiate into ecto-, endo- and mesodermal germ layers, we promoted the spheroid formation, as it is well known that by three dimensional spheroid culture a selection of higher potent cells can be selected and let therefore UC-MSCs outgrown on chitosan coated surfaces. Chitosan, a de-acetylated derivate of chitin, is a positive charged polysaccharide and well established to promote spheroid formation of adherent cells. As a result, the chitosan coating leading to a higher count of smaller spheroids than uncoated surfaces and approximately 20 spheroids with a diameter between 80 and 100 and 40 spheroids between 100 and 200 μm could be obtained from one donor after 33 days of cultivation. (data not shown.) For further multiplication and selection, the “chitosan” spheres (Fig. 5a, left) were seeded on a collagen-coated surface. The spheres grew out on collagen and as illustrated in Fig. 5a, left, the VSELs deposited on top of the confluent cell layer which show a hill and valley morphology reminiscent of a smooth muscle cell association. However, without taking care of the sticking population of the tiny cells the adhering cell layer was trypsinized and seeded onto matrigel coated glass slides and afterwards treated with appropriate induction media to determine the tri-lineage differentiation potential. It was noticeable that despite discarding supernatants containing VSELs the proportion of tiny cells were immediately visible again after 24 h of cultivation, (Fig. 5a, right). Moreover, despite daily induction-medium exchange, the population of the tiny cells were still available after 4 and 7 days of cultivation. After seven days of cultivation the morphology of the most cells did not really look different from each other although stimulated with the different induction cocktails. Immunofluorescence analysis demonstrated that only the tiny cells and cells that surrounded in immediate vicinity the VSEL accumulations were double stainable for ectodermal associated transcription factor Sox-2 and Pax6 (Fig. 5b, top). For endodermal induction mostly all cells were positive for CD184 but only the tiny and their adjacent cells also positive for Sox-17. The single CD184 chemokine receptor staining is for the identification of endodermal differentiation induction not meaningful as this marker is also expressed by cells of ecto- and mesoderm (Fig. 5b, middle) [32, 33]. As a result, only the double stained cells can be classified as cells of early endodermal inducted cells which are only visible in regions of higher VSEL density. For mesodermal induction expression of surface receptor CD144 and CD140b were stained. It was noticeable here that the cell number decreased considerably due to mesodermal induction and only a few cells remained for staining at the end. (Fig. 5b). However, the staining of mesodermal marker CD144 and CD140b were detectable for nearly all remaining cells. (Fig. 5, bottom).

## Discussion

The aim of this study was to investigate the potency of UC-MSCs for their respective expression of pluripotency-associated markers and tri-lineage differentiation potential. From the beginning, therefore, we focused on cells with typical morphology of pluripotent stem cells (PSC) at an early developmental stage, which are known to have a small and round shape with large nuclei surrounded by a thin rim of cytoplasm enriched with spherical mitochondria [34]. Despite simple 2D cultivation conditions known to be unfavorable to retain stem cells in a multipotent state [35], we always observed a weak appearance of small round cells of approximately 5 to 7 μm, and hypothesized that this population might cause the detection of pluripotency-associated marker expression.

In the literature, we noted that pluripotency-indicating signals could be measured to different levels dependent upon the individual experimental setup. In one elegant study, Carlin et al. presented [20] a clearly upregulation of *OCT4*, *NANOG* and *SOX2* genes of porcine-derived umbilical cord tissue cells cultured in FCS-containing media to which EGF and FGF were added.

Fong CY [22] investigated the effect of embryonic stem-cell media on human WJ MSCs and could measure pluripotency-associated cell surface markers and, in comparison to embryonic stem cells, a weak gene expression for pluripotency-associated transcription factors.

Other groups added knock-out serum to FCS-containing media and gained minimal expression enhancement of different pluripotency-associated markers [36]. Karahuseyinoglu et al. [37] confirmed that UC-MSCs cultured in DMEM with 10 % FCS can be differentiated into mesodermal lineage cells that can express neuronal markers through biochemical neuronal induction.

However, among all the studies, Carlin et al. was one of the few who noticed small round cells that formed colony forming units and emphasized that these cells might cause the measured pluripotency. Moreover, he explained that these cells may be descendants of migrated primordial germ cells (PGCs). PGCs are the founder cells of sperm and egg, and have the unique ability to generate a totipotent zygote at fertilization; they derive from posterior epiblast, and also from extra-embryonic amnion according to recent studies [38].

After induction, primate PGCs expand in numbers, enter the yolk sac during gastrulation [39] and reside, according recent studies with monkey embryos [40], within the posterior side of the yolk sac at the base of the connecting stalk and the incipient allantois [40]. To ultimately reach their final destination, PGCs then translocate actively by migration, or passively due to their local proximity, according to the latest research [41], from within the hindgut into the dorsal mesentery towards the developing gonadal ridges [41]. However, independent of their original deposition and their way to gonadal riches, it is likely that remnants of PGCs and or their descendants remain in regions of the umbilical cord, as on day forty after fertilization, while the yolk sac and the allantois elongate into the connection stalk and are compacted into a narrow cord covered with amniotic epithelium, which forms the umbilical cord [42]. Ratajczak et al. hypothesized that PGCs might be the founder cells of so-called very small embryonic stem cells (VSELs) [34, 43] as they express several markers of migrating PGCs, such as embryonic stem cell markers, like Nanog, Oct-4, SSEA-4, Rex-1 and also germ-line specific markers such as Stella and Fragilis. Furthermore, VSELs are like PGCs: quiescent [44] and stress resistant [45], but can be expanded *in vivo* in response to stimulation by pituitary gonadotropins and gonadal sex hormones. In contrast to embryonic stem cells, VSELs are not able to complement blastocyst development or teratomic formation. *In vitro* RT-PCR and immunofluorescence analysis demonstrated that, in comparison to murine embryonal stem cell line (ES), primary murine-bone marrow (BM) derived VSELs expressed *NANOG* and *OCT4* more strongly and have the capacity to differentiate into three germ layers [29]. Until now, it is well established that VSELs reside in human BM, gonads and umbilical cord blood [43].

We are the first group postulating that VSELs also exist in WJ tissue. They not only have the same small size, 5 to 7 μm, but also the typical appearance of luminescent cells with dark points and the well-known floating behavior so that the majority of VSELs can be found in the supernatant after moderate centrifugation of WJ-MP. Taking into account that Kucia et al. could demonstrate that C2C12 murine myoblastic cells promote VSELs proliferation and sphere formation [29], we suspected that long-term cultivation of WJ-MP could also function as a feeder layer because in previous studies we could demonstrate that a large proportion of long-cultured WJ-MP differentiated more into the myoblastic-like lineage cells (data not shown).

However, instead of determining the decisive contents of the myo-fibroblastic/myoblastic feeder supernatants, we observed the effect on VSELs duplication and sphere formation behavior. Although this feeder-based method is unsuitable for producing high amounts of VSELs, we gained enough cells for comprehensive characterization procedures.

Without sorting, but with specific centrifugation and filtering processes, we separated the round small-sized cells from the WJ-MP and could stained pluripotency-associated transcription factors and VSEL typical surface markers CD133, CXCR4, SSEA-1 SSEA-4 with flow cytometry. At this point it became clear that our separation technique was unsuitable, as through the frequent centrifugations steps we produced a lot of cell debris of small size, which unspecifically bound antibodies and generated the high background signal. Nevertheless, we were indeed able to isolate enough cells that show – similar to embryonic stem cells – a 10- to 1000-time lower expression rate of pluripotency marker coding genes [46], but show, in relation to the negative controls, a normalized gene expression of more than 100. Furthermore, immunofluorescence analysis confirmed the existence of pluripotent VSEL-like cells, and, in accordance to others, we could demonstrate that the distribution of markers varied for each sample (data not shown). We could hypothesize that this might be dependent on the origin and differentiation stage of the individual cells [47].

However, despite the detection of VSEL-like cells, we do not exclude other cell sources within the mixed WJ-MP that also exhibit pluripotent properties. This may be due to the small round or triangular cells that have also been sighted in the WJ-MP and that detach from adherent cell layers after some point and form spheres. Furthermore, it cannot be ruled out that VSELs are part of spheres of different cell populations.

We demonstrated that slight amounts of VSELs are always dragged along as we could detect their existence in cultures, also after the passaging or freezing of the WJ-MP population, although supernatants were discarded after low centrifugation speed (supplemental data). Moreover, the VSELs were preserved even after various forms of cultivation, e.g. they were not lost through chitosan-stimulated WJ-MP spheroid formation, as the tiny cells were clearly present after outgrowth of “Chitosan performed spheroids” on collagen-coated surfaces. While the stimulus of collagen and FCS-containing medium was sufficient to direct the out-grown cells of WJ-MP spheroids further into myogenic morphology (supplemental data), the VSELs remained unaffected by this differentiation influence and could be easily recognized on the surface of the WJ-MP culture. After collagen cultivation the WJ-MP were again detached (low centrifugation speed led to VSEL loss) before seeding on matrigel coated glass slides to detect their potential for tri-lineage differentiability. Despite a high cell-seeding concentration, the cell density of large WJ-MP cells was reduced after incubation in induction media, after 24 h, while the presence of VSELs were again apparent for each of the three tri-lineage samples. In addition, we could not detect any distinct morphological change in the large adherent cells, speculating that the biochemical inducers do not target opened promoters of them. However, a few cells seemed to survive the induction procedures and as they are in local proximity to single VSELs one could hypothesize that these emerged from them.

In summary, we could demonstrate that a very small population of WJ-MP cells disposes of pluripotent stem-cell characteristics and claim that a part of these cells are VSEL-like cells. In addition, we could demonstrate that adherent UC-MP with their myo-fibroblastic characteristics could be used as feeder to preserve the VSELs for longer cultivation times. Future analysis should be made to shed more light on their particularities and to present their value for regenerative medicine purposes.

## Materials and Methods

### Cell Isolation from UC

The umbilical cords were received from Universitätsklinikum RWTH Aachen after obtaining the informed consent of the donor’s parents. Once in the laboratory, the UC was cleaned under the running tap, residual blood was removed. Under sterile conditions the UC was disinfected in 70 % ethanol for 30 s and rinsed three times with Dulbecco’s phosphate-buffered saline (PBS), supplemented with 500 U/mL penicillin and 500 µg/mL streptomycin. The UC was cut into small pieces with a length of about 1–2 cm. To avoid contamination of the subsequent cell culture with blood cells, areas that contained large blood clots or were strongly twisted were discarded and arteries and veine were carefully pulled out. For cell outgrowth the UC pieces were stick with the Wharton´s Jelly side to the petri dishes and covered with DMEM, supplemented with 15 % heat-inactivated fetal bovine serum (FBS), 100 U/mL penicillin, 100 µg/mL streptomycin, 2.5 µg/mL Amp B and 0.1 % CFA. After 10–15 days of incubation at 37 °C in 5 % CO_2_, the first cells migrated out of the tissue pieces. The first medium change took place after 5 days and was carried out every 2–3 days. The tissue pieces were discarded after the outgrowing cells had reached a 50 % confluence.

### Cell Culture

When UC-MSCs reached confluence of approximately 80 % the cell culture was split for cell expansion. For passaging adherent cells were washed with PBS^−/−^and detached with Trypsin/EDTA. The trypsin reaction was competitively inhibited by adding four times the amount of culture medium (CM) containing DMEM, supplemented with 10 % heat-inactivated fetal bovine serum (FBS), 100 U/mL penicillin, 100 µg/mL streptomycin, 2.5 µg/mL Amp B and 0.1 % CFA.

### Concentration of VSEL Cells

For up-concentration of VSELs, cell culture supernatants were centrifuged with 195xg to pellet dead cells and heavier, voluminous protein fragments. After, one third of the supernatants was re-pipetted into the cell culture flask of the freshly passaged cells. This process was repeated 3 to 4 times until the VSELs were extracted from the supernatants by a comprehensive medium reduction protocol: Briefly, the supernatants were divided up into many 2ml Epis and centrifugated by 300xg for 10 min. The resulting pellets were filtered with minimal amount of medium through filters with a pore size of 7 μm and the filtrate was used for further analysis.

### FACS

Flow cytometry measurements were performed at the University Hospital Aachen, Germany. Analysis was exerted on a FACS Canto II (Becton Dickinson, USA). For FACS analysis VSELs were washed with PBS and counted with a hemocytometer and the whole cell amount were divided into reaction tubes for each antibody or antibody pair and for each isotype control with a concentration of up to 0.3 × 10 ^6^ cells.

For fixation, permeabilization and staining procedures buffers by “Transcription Factor Staining Buffer Set” by Miltenyi Biotec (Berglisch Gladbach, Germany) were used. First surface antigens CD133 (1:50, Miltenyi), CD184 (1:50, Miltenyi), SSEA-1 (1:50, Miltenyi) and SSEA-4 (1:500, Thermo Fisher Scientific) were stained after blocking with Fc blocking solution (Miltenyi) at RT for 1 h. After, cells were washed with (PBS with 2 %BSA) washing buffer, centrifugated at 600xg for 10 min. Afterwards supernatants were aspirated, cells were resuspended in Fixation Permeabilization solution and incubated at 4 °C for 30 min. After further washing steps antibodies against antigens of nuclei located transcription factors namely Oct-4 (1:40, Thermo Fisher), Sox-2 (1:200, Invitrogen) and Nanog (1:50 Miltenyi) were added and incubated for in the dark at 4 °C for 30 min. After three times washing with washing buffer cells were re-suspended in 1ml PBS / sample. Before flow cells were measured by flow cytometry, nuclei of each sample were stained with HOECHST dye.

### RNA Isolation of VSELs Spheroids and MSC

Cell pellets were rinsed twice with PBS^−/−^ and 0.4 mL precooled TRIzol™ Reagent and the cell lysate was collected with a P1000 pipette tip and transferred to a 1.5 mL reaction tube. The lysate was homogenized by repeated pipetting up and down. For dissociation of nucleoprotein complexes, the lysate was incubated for 5 min and 80 µL chloroform were added. The samples were incubated for another 2–3 min and centrifuged at 12,000 g and 4 °C for 15 min to allow the homogenizate to separate into a clear upper aqueous layer, containing the RNA, an interphase and a red lower organic layer, containing the DNA and proteins. Due to the low initial volume of the sample, 10 µg RNase-free glycogen were added to the aqueous phase a carrier. The glycogen was co-precipitated with the RNA but did not interfere with later applications. Subsequently, 200 µL isopropanol were added to the aqueous phase, incubated for 10 min, and centrifuged for 10 min at 12,000 g and 4 °C. The RNA precipitate formed a pellet at the bottom of the tube, the supernatant was discarded, and the pellet was washed in 400 µL ethanol (75 %). The sample was gently mixed and centrifuged for 5 min at 7,500 g and 4 °C. The supernatant was aspirated, and the RNA pellet was air dried for 10 min and resuspended in 20 µL RNase-free water. The RNA quality and yield were determined by ultraviolet-visible spectroscopy (UV-VIS) with the Implen NanoPhotometer® P330 (Implen, Munich, Germany) using a lid factor of 10 and a sample volume of 1 µL. The RNA was either proceeded immediately in downstream applications or stored at -80 °C.

### cDNA Synthesis

cDNA synthesis was performed using the iScript™ Select cDNA Synthesis Kit (Bio-Rad Laboratories GmbH, Feldkirchen, Germany) according to the manufacturer’s instructions.

### RT PCR

The quantitative real-time PCR assay was performed using the SsoAdvanced Universal SYBR® Green Supermix and the PrimePCR SYBR® Green Assay (Bio-Rad Laboratories GmbH, Feldkirchen, Germany). Both the products and the cDNA samples were thawed to room temperature, thoroughly mixed by inversion, and briefly centrifuged to collect the solutions at the bottoms of the tubes. All work was carried out on ice and protected from light. For each primer, 20 µL of master mixture was prepared containing 1µL 20x PrimePCR Assay (Primer), 10 µL 2x SsoAdvanced Universal SYBR® Green Supermix and 8 µL nuclease-free water with 1 µL cDNA sample finally being added. Each reaction mixture was prepared in triplets for each sample in a 96-well PCR plate, which was sealed before being loaded into the CFX 96 Touch™ Real-Time PCR Detection System (Bio-Rad, Feldkirchen, Germany). The applied thermal cycling protocol consists of an initial 2 min activation step at 95 °C followed by both 40 denaturation (95 °C for 5 s) and annealing (60 °C for 30 s) steps. The plate was read after each annealing step and the melting curve was measured in one heating phase from 65 to 95 °C (0.5 °C increments with 5 s per step).

Sample analysis was performed using the software ‘Bio-Rad CFX Maestro 1.1, Version 4.1.2433.1219’ (Bio-Rad Laboratories, Inc., Hercules, California, USA), which refers to Pfaffl, 2001 [305] and Vandesompele et al., 2002 [306]. The house keeper genes (HKG) Glyceraldehyde 3-phosphate de-hydrogenase (GAPDH) and Beta-2-Microglobulin (B2M) were used to normalize gene expression of the different genes of interest (GOI).

### Immunofluorescence

Immunofluorescence measurements were performed with Fluorescence Microscopy BZ-II Analyzer (Keyence, Frankfurt am Main, Germany). For immunofluorescence staining, VSELs were washed with PBS and were used for staining procedures after fixation with 4 % paraformaldehyde for 30 min at 4 °C. After fixation, cells were centrifuged at 600 g for 5 min, washed three times in PBS and blocked with 10 % normal goat serum and 2 % BSA in PBS for 60 min. For antibody staining, cells were aliquoted in reaction tubes and incubated with primary antibodies against CD133 (1:50, Miltenyi), CD184 (1:50, Miltenyi) and SSEA-4 (1:500, Thermo Fisher Scientific) or against appropriate isotype control antibodies overnight, at 4 °C. Next day, cells of each aliquot were washed three times with PBS /0.1 %BSA cells and stained with secondary antibody for 30 min at room temperature. For nuclear staining, cells were lastly washed twice for five minutes with PBS / 0.1 %BSA, after centrifugation at 600 g the cell pellets were re-suspended in HOECHST containing PBS (1:10).

For intracellular nuclear staining of transcription factors Nanog, Oct-4, and Sox-2 “Transcritption Factor Buffer Set” by Miltenyi Biotec was used and performed according to manufactor´s protocol. Briefly, PBS supernatants of washed and centrifuged VSEL aliquots were aspirated and cells were fixed for 30 min at 4 °C in 500 µl cold fixation/permeabilization solution. After, centrifugation at 600 g the pellets were re-suspended with blocking solution consisted of permeabilization buffer with 2 % BSA and 10 % normal goat serum and incubated at 4 °C for one hour. Afterwards, cells were intracellular nuclear stained with primary antibody against Oct-4 (1:50, Miltenyi), Sox-2 (1:50, Miltenyi) and Nanog (1:50 Miltenyi) or with appropriate isotype controls at RT for 1 h. In a last step cells were washed twice with permeabilization buffer and subsequently nuclei were stained with HOECHST dye.

### Trilineage Differentiation

The trilineage differentiation potential of spheroid derived stem cells was investigated.

Sphäre formation was promoted by outgrowth of UC-tissue on chitosan coated vessel surfaces. For coating 0.01 g/ml chitosan was diluted in 0.76 % acetic acid and 2.5 ml solution were applied per 10 cm^2^ surface area. After evaporation of the solution the reaction was neutralized with an equivalent of 0.5 N sodium hydroxide for 2 hours. After washing with deionized sterile water, the surfaces were sterilized with UV-light for 12 hours. Umbilical tissue pieces were outgrown on the chitosan coated vessels and generated spheroids were taken after 44 days. In order to expand the spheroid containing cells the spheroids were outgrown on collagen 1 coated surfaces. For collagen from rat tail were used (Life Technologies) and applied in accordance to the instruction guidelines. To investigate the tri-lineage differentiation potential the on collagen 1 outgrown UC-MSCs were detached by trypzination after reaching 80 % confluence and seeded on matrigel (Corning) coated coverslip. Immediately after seedeing the adhering cells were incubated with in” StemMACS ^TM^ ecto-, meso- and endo-diff Medium” according to the instruction guidelines (Miltenyi Biotec, Bergisch Gladbach, Germany). The medium was changed daily, and the cell morphology was detected by phase contrast microscopy (Axiovertt 40 CFL, Carl Zeiss Microscopy, Munich, Germany). On the day seventh the differentiation into three lineages were assessed immunocytochemically by fluorescence microscopy (Biorevo BZ-9000, Keyence, Neu-Isenburg, Germany), regarding the expression of the ectodermal progenitor marker proteins Sox-2 and Pax6, the expression of the endodermal early marker Sox-17 and CD184, and the expression of mesodermal marker CD144 and CD140b. For the detection of nuclei, cells were stained at least with Dapi 1:10.000 in PBS -/- for 10 min.

## Conclusions

We were able to demonstrate the presence of VSELs in Wharton´s Jelly tissue. The existence of this pluripotent stem cell source and the possibility to use the WJ MP as feeder layer enable to keep them from getting lost and to receipt their pluripotency characteristics.

## Supplementary Information

ESM 1 VSELs were incubated with Mouse IgG control or with REA Control (S)-FITC to analyze the unspecific staining for SSEA-4 (c) or for CD184 and CD133 (a) respectively.  As the nuclear located transcription factor proteins Nanog, Sox-2 and Oct-4 are coupled to the same recombinant human IgG1, the same isotype control namely (REA (I) –PE) were used (b). Nuclei were stained with HOECHST dye. No fluorescence signal could be observed. The scale bars represent 50µm. (JPG 43.9 KB)

ESM 2 Isotype controls for all tri-lineage differentiation markers. For isotype controls staining UC MP cells were incubated with REA-Control-PE and REA Control-FITC to analyze unspecific staining of meso- and endodermal marker. For ectodermal isotype control Isotype control REA Control PE and Rat IgG2a, k was used. Nuclei were stained with DAPI. No fluorescence signal could be observed. The scale bars represent 50µm (a). For b) and c) the same magnification were used as for a). (JPG 96.8 KB)

## Data Availability

All authors ensure that all data and materials support the published claims and comply with field standards.
