# Supplementary figures and images for: The Treasury of Wharton's Jelly
Source: Stem Cell Rev Rep. 2021 Oct 13;18(5):1627–38. doi: 10.1007/s12015-021-10217-8 (PMC9209346; doi:10.1007/s12015-021-10217-8)

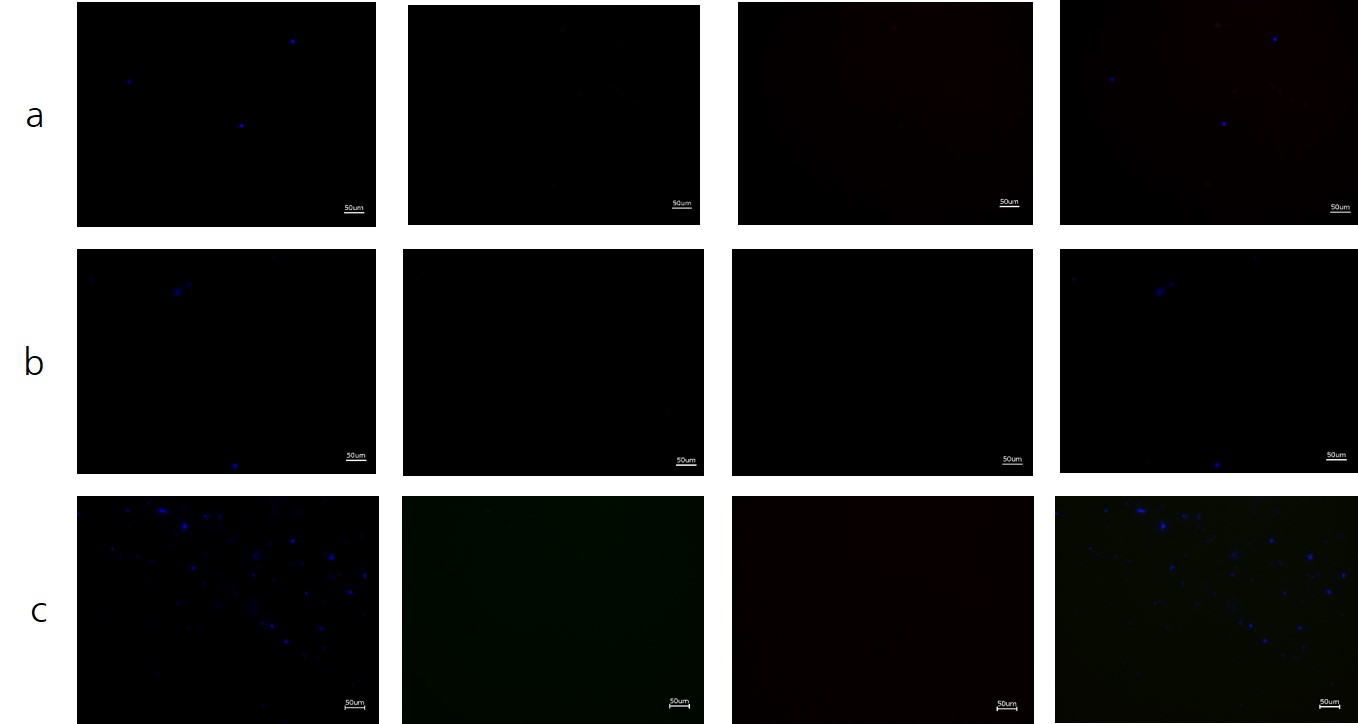

Supplement: Supplementary file 1 — VSELs were incubated with Mouse IgG control or with REA Control (S)-FITC to analyze the unspecific staining for SSEA-4 (c) or for CD184 and CD133 (a) respectively. As the nuclear located transcription factor proteins Nanog, Sox-2 and Oct-4 are coupled to the same recombinant human IgG1, the same isotype control namely (REA (I) –PE) were used (b). Nuclei were stained with HOECHST dye. No fluorescence signal could be observed. The scale bars represent 50µm. (JPG 43.9 KB) [file 12015_2021_10217_MOESM1_ESM.jpg]

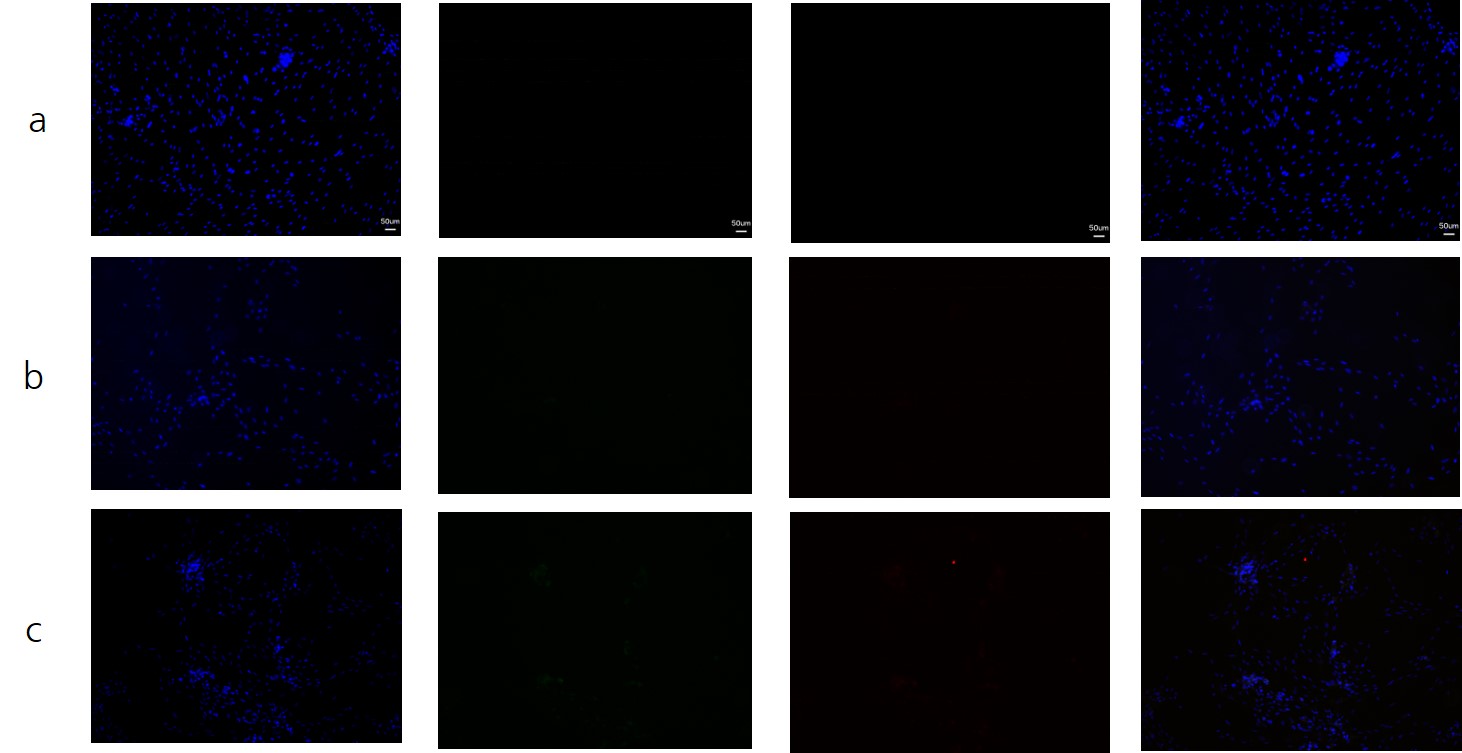

Supplement: Supplementary file 2 — Isotype controls for all tri-lineage differentiation markers. For isotype controls staining UC MP cells were incubated with REA-Control-PE and REA Control-FITC to analyze unspecific staining of meso- and endodermal marker. For ectodermal isotype control Isotype control REA Control PE and Rat IgG2a, k was used. Nuclei were stained with DAPI. No fluorescence signal could be observed. The scale bars represent 50µm (a). For b) and c) the same magnification were used as for a). (JPG 96.8 KB) [file 12015_2021_10217_MOESM2_ESM.jpg]
